# A humoral diagnostic test outperforms cellular tests in a farm with a latent tuberculosis outbreak caused by a new *Mycobacterium tuberculosis* complex spoligotype that affected sheep but not goats

**DOI:** 10.3389/fvets.2023.1310205

**Published:** 2024-01-22

**Authors:** Ramon A. Juste, Leire Fernández-Veiga, Miguel Fuertes, Ignacio Fernández-Ortiz de Murua, Guillermo Cardona, Maria V. Geijo, Joseba M. Garrido, Iker A. Sevilla

**Affiliations:** ^1^Animal Health Department, NEIKER – Basque Institute for Agricultural Research and Development, Basque Research and Technology Alliance (BRTA), Derio, Spain; ^2^Servicio de Ganadería, Diputacion Foral de Alava, Vitoria-Gasteiz, Spain

**Keywords:** sheep tuberculosis, goat tuberculosis, SB2737, *Mycobacterium caprae*, tuberculosis antibody test, bovine tuberculosis, skin test

## Abstract

Tuberculosis (TB) is a disease caused by members of the M. tuberculosis complex (MTC) that affects numerous species. M. caprae, a member of the complex which is close to M. bovis, is emerging and affects several different hosts that include goats, cattle, sheep, pigs, rabbits, wild boar, red deer, foxes and also humans. A new M. caprae spoligotype (SB2737) was isolated from an outbreak of sheep tuberculosis affecting a mixed sheep (323)-goat (29) farm in 2021. The index case was detected by the La Rioja slaughterhouse veterinary inspection. Tracing back to the farm of origin, both species were submitted to Comparative Intradermal Tuberculin Test (CITT) and M. bovis-specific antibody ELISA tests. A subsample was also examined by IFN-γ release assay (IGRA) and all positives were slaughtered and pathologically and microbiologically investigated. Only 1.2% of sheep and no goat were positive in the CITT, and 11.4% in the IGRA sheep subsample, while up to 36.8% were positive in two consecutive M. bovis-specific antibody ELISA tests. Goats had always tested negative in annual intradermal follow-up since 2013. Upon confirmation of the immunologically positive sheep at slaughter, all the remaining negative animals were killed and 29.2% of sheep were still found infected. This raised the final overall prevalence to 37.5%. Antibody ELISA was the most sensitive (81.4%) in vivo detection method still showing a 85.0% specificity relative to pathological and microbiological tuberculosis status. It was nearly 10 times more sensitive than skin test and had an 86.8% positive predictive value. Notwithstanding a possible singular pathogenesis of the new spoligotype, this outbreak adds up to previous reports suggesting that sheep tuberculosis could be huge reservoir of infection worldwide overlooked by skin test low sensitivity or simply lack of investigation. This makes it urgent to extend the use antibody tests to address the Trojan horse of hidden M. tuberculosis complex infections on bovine TB control programs.

## Introduction

Tuberculosis (TB) is a disease caused by members of the *M. tuberculosis* complex (MTC) that affects numerous species. The two more common agents are *M. tuberculosis* itself, which affects mainly humans, and *M. bovis,* which affects many different ruminant and non-ruminant species. However, infection with *M. caprae*, another member of the complex that is close to *M. bovis*, is emerging and affects several different hosts including goats, cattle, sheep, pigs, rabbits, wild boars, red deer, foxes, and also humans (1–4). Spoligotyping has proven very useful to characterize the different variants for epidemiological studies and to link outbreaks. Given its impact on human health and the connection of bovine TB with human cases, especially digestive forms in children caused by transmission through milk, eradication of the infection from cattle was soon undertaken in many countries. The success of the programs designed to identify and cull infected cattle through subclinical infection detection made them compulsory in many countries and have defined the standards for international animal health policies (5). Since the main focus of these programs was to prevent milk transmission of MTC, their objective has been almost exclusively the bovine species, which is the main source of milk for human consumption. The lower risk associated with meat transmission, the smaller individual value, and the restricted market of small ruminant milk has attracted much less attention to TB control in goats and sheep, and therefore, these species have not been included in TB eradication programs until very recently, and even so, only in relation to cattle TB outbreaks (6, 7). Although the role of goats was more widely acknowledged, sheep had long been considered resistant to tuberculous infections (8). Therefore, the disease was considered extremely rare in this species (9, 10) and sheep is not even mentioned by the WOAH Terrestrial Code with regard to tuberculosis (11). However, recent studies have shown that the species is fully susceptible and is often infected with MTC mycobacteria in bovine and caprine mixed farms where TB has been detected (7, 12–17). Here, we report a case of MTC infection in a sheep-goat mixed farm whose index case was detected by passive slaughterhouse surveillance and where standard cellular tests failed to detect the vast majority of infected individuals, but a humoral multispecies test showed a high sensitivity. We think that these case features, sheep-only outbreak, new spoligotype, and humoral test outperformance of cellular ones, are novel features that, contributing to the depiction of a more complex model of bovine TB (18), must be taken into account to make animal TB control a reachable objective in the not-too far future.

## Materials and methods

**Figure 1:**
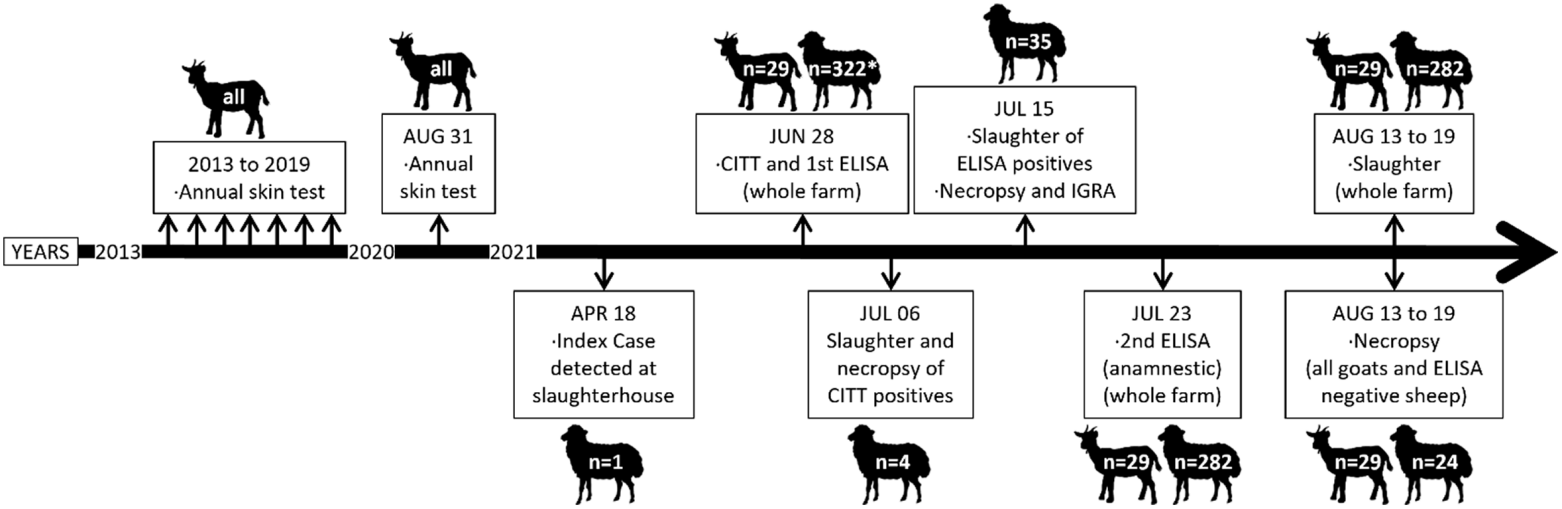
Chronological overview of the events and actions undertaken in relation to the tuberculosis outbreak detected in the goat-sheep mixed farm. *One ewe had only a record for CITT on 28 June control.

Lesions compatible with TB were detected in the routine slaughterhouse inspection (Slaughterhouse of Logroño, La Rioja, Spain) of a culled ewe coming from Laguardia (Basque Country, Spain) on 18 April, 2021 (see the timeline presented in Figure 1). Samples from lesions were sent to the European Union Reference Laboratory (EU-RL) for Bovine TB (VISAVET-Universidad Complutense de Madrid) for MTC culture, and *M. caprae* was isolated. Spoligotyping yielded a new pattern that was registered at the Spanish MycoDB database with code SB2737. Regional animal health authorities immediately checked that the goats in the flock had been annually skin test (ST) negative to TB since the caprine TB eradication program began in 2013 (the last control on 31 August 2020). There was no history of paratuberculosis in the flock, and paratuberculosis vaccination had never been used. Then, a prevalence investigation that included a comparative intradermal tuberculin test (CITT) and an antibody ELISA (INgezim Tuberculosis DR; INGENASA-Eurofins, Gold Standard Diagnostics) (19) on the whole flock (321 sheep and 29 goats) was ordered and performed on 28 June 2021. The ST was performed and interpreted following the EU-RL guidelines published by the Spanish Ministry of Agriculture, Fisheries and Food (MAPA), which are in agreement with the regulations of the European Union (Commission Delegated Regulation 2020/689 of 17 December 2019). Goats and sheep were administered 0.1 mL PPD-B (2,500 IU) and PPD-A (2,500 IU) (CZ VACCINES, Pontevedra, Spain) through separate intradermal injections in the neck using a Dermojet (Akra Dermojet, Pau, France). The skin thickness was measured before and 72 h after inoculation. Interpretation was done for both single intradermal tuberculin test (SITT) and comparative intradermal tuberculin test (CITT). An animal was considered SITT positive, inconclusive, or negative when the increase of the PPD-B site was 4 mm or greater, between 2 mm and 4 mm, or less than 2 mm, respectively. For CITT, animals were considered positive, inconclusive, or negative if the increase of the PPD-B injection site exceeded that of the PPD-A site by greater than 4 mm, 1–4 mm, and less than 1 mm, respectively. The antibody ELISA was run and interpreted according to the supplier instructions. At 72 h ST reading, only three ewes and one ram were positive to the ST (according to both SITT and CITT interpretation) and were sent to NEIKER-BRTA laboratories for necropsy on 6 July 2021. Antibodies against *Mycobacterium bovis* MPB83 protein (conserved also in *M. caprae*) were detected by ELISA in 41 sheep (37 positive and 4 doubtful), including three out of the four ST-positive individuals. TB-characteristic lesions were confirmed, and SB2737 *M. caprae* was isolated from all four necropsied sheep. Subsequently, the remaining sheep (except three) with positive or doubtful ELISA result (*n* = 35) were slaughtered (15 July 2021), and blood was collected for interferon-γ release assay testing (IGRA). For IGRA, sodium heparin whole blood was distributed into cell culture plates (1.5 mL/well) and stimulated with PPD-B, PPD-A (both PPDs at 20 μg/mL) and phosphate-buffered saline (PBS; no stimulation) in individual wells (no mitogen was included), starting within 4 h of blood collection. After overnight incubation at 37°C in 5% CO_2_ atmosphere, samples were centrifuged and supernatants analyzed using the MAPA validated IDScreen® Ruminant interferon-gamma ELISA kit (IDvet, Grabels, France) following manufacturer’s instructions. Results were interpreted according to the indications of the kit (positive cutoff: s/*p* ≥ 16). During necropsy, all tissues were macroscopically inspected, and samples were collected for histopathological (lung, liver, spleen, intestine, pancreas and mediastinal, tracheobronchial, tracheal, hepatic, mesenteric, ileocecal, and ruminal lymph nodes) and microbiological (mediastinal lymph node) analysis. *M. caprae* infection was confirmed in all of them except for three (one of the ELISA doubtful and two ELISA positive). Given the high seroprevalence detected with the antibody ELISA and the high infection confirmation rate among positives, an anamnestic antibody ELISA was performed (23 July). In view of the increased seroprevalence detected with the anamnestic ELISA, the whole flock including goats was sent to the slaughterhouse between 13 and 19 August 2021. This time 24 out of the 200 sheep displaying a negative result in both ELISA tests, and all goats (*n* = 29) were submitted to necropsy and MTC isolation. Gross inspection of all tissues and microscopic examination of lung and mediastinal and tracheobronchial lymph nodes were carried out. Samples from tissues with lesions or a pool of the tissues specified above if lesions were not present were submitted to MTC isolation culture. TB infection was confirmed if TB lesions were observed and/or *M. caprae* was isolated. Thus, a total of 93 animals (64 sheep and 29 goats) were necropsied and had pathological and microbiological results in addition to *in vivo* tests results.

The epidemiological investigation included collection of information to determine management, replacement rates, origin of purchased animals, possible contacts with other farms, and recent TB outbreaks in the municipality. Drinking points in the grazing area were investigated for MTC by isolation culture.

Fisher’s exact probability test (FET) was used for comparison of frequencies (20). A complementary sensitivity was calculated as the percent of positive to one test and negative to the reference over the total number of positives in the reference test. This figure gives a measure of how much a test outperforms the reference to which it is compared (21). A generalized linear model and a correlation analysis were carried out to assess the association of age with optical density (OD) of immune tests using the jamovi statistical software (20, 22).

## Results

Table 1 shows a summary of the animals according to different grouping criteria and test results. A total of 351 animals (322 sheep and 29 goats) were processed at different times and with different methods (see Figure 1). Four breeds of sheep were present in the farm (70.9% Navarrese breed). About two-thirds were breeders 24 months or older. Only 1.2% reacted positive in the intradermal test. In contrast, there were about 10 times more positive animals (12.8%) in the first ELISA testing and twice as many (28.4%) in the anamnestic ELISA. Jointly, the ELISA identified 37.27% antibody-positive sheep. The infection was confirmed in as much as the 94.6% (35/37) of positive/doubtful individuals of the first ELISA. The IGRA was applied only to 35 of the 41 antibody ELISA-positive/doubtful sheep and was positive only in 4 of them. In total, TB was pathologically or microbiologically confirmed in 44 out of the 64 sheep investigated (68.75%), including the index case. However, of these, 24 had been randomly selected at slaughter among test negative ones. Since seven of them (29.2%) were also TB confirmed, it can be estimated that there were about 77 additional infected sheep that had been missed. This would make the overall prevalence in the sheep flock reach 37.5% (121 out of 323). The age of these randomly selected animals was significantly lower than the age of the rest (*p* = 0.0187). Age was positively correlated with OD readings in the anamnestic ELISA (*r* = 0.2235, *p* = 0.0002) and its increase (*r* = 0.1292, *p* = 0.0455), but not with OD readings in the first antibody ELISA (*r* = −0.0880, *p* = 0.1418), or bovine antigen in IGRA (*r* = −0.0483, *p* = 0.7830).

**Table 1:** General results in sheep.

	Level	Overall			Tuberculosis +		
		Count	Total	Proportion	Count	Total	Proportion
Breed	Chamarita	6	319	0.0188	0	0	−
	Churra	12	319	0.0376	1	42	0.0238
	Navarrese	226	319	0.7085	33	42	0.7857
	Mixed	75	319	0.2351	8	42	0.1905
Sex	Female	306	319	0.9592	40	42	0.9524
Age	Ewe/Ram	246	319	0.7712	38	42	0.9048
ELISA1	+	41	321	0.1277	35	43	0.814
ELISA2	+	80	282	0.2837	0	0	−
ELISA1&2	+	120	322	0.3727	35	43	0.814
CITT	+	4	322	0.0124	4	43	0.093
IGRA	+	4	35	0.1143	4	32	0.125
Isolation	+	40	63	0.6349	40	43	0.9302
TBLES	+	39	63	0.6190	39	43	0.907
Lung	+	24	63	0.3810	24	43	0.5581
Lymph node	+	42	63	0.6667	42	43	0.9767
Viscera	+	10	36	0.2778	10	33	0.303
Tuberculosis	+	43	63	0.6825	43	43	1

Overall, Absolute and relative counts of positive over total examined sheep with each method; Tuberculosis +, Absolute and relative counts of positive over pathologically or microbiologically tuberculosis-confirmed sheep with each method; ELISA 1, 2, 1&2, Anti-*M. bovis* specific antibody test in each round and in combination; CITT, Comparative intradermal tuberculin test; IGRA, IFN-γ release assay; TBLES, Tuberculous lesions; Lung, Tuberculous lesions in lungs; Lymph node, Tuberculous lesions in lymph nodes; Viscera, Tuberculous lesions in abdominal viscera; Tuberculosis, Post-mortem pathologically or microbiologically confirmed tuberculosis.

The diagnostic method performance showed large differences regarding sensitivity (Table 2). While both standard cellular tests had a low sensitivity at around 10%, the ELISA had over 80% sensitivity. These figures led to a high outperformance of the ELISA relative to the cellular tests (over 700%), which, however, only had a 7% complementary sensitivity relative to the post-mortem tuberculosis status.

**Table 2:** Performance of the tests.

	*N*	Prevalence (*n*)	Se	Sp	Kappa (p)	Complementary Sensitivity				+ Predictive
						Tuberculosis	CITT	IGRA	ELISA	
CITT	322	1.2% (4)	9.3%	100%	0.0611 (0.1587)	0.0%	-	0.0%	2.4%	
IGRA	35	11.4% (4)	12.5%	100%	0.0239 (0.5153)	0.0%	0.0%	-	0.0%	
ELISA 1	321	12.8% (41)	81.4%	85.0%	0.6223 (<0.0001)	7.0%	950.0%	775.0%	-	86.8%
ELISA 2	282	28.4% (80)								
ELISA 1&2	321	36.8% (118)								
Pathology	63	61.9% (39)	90.7%	100%	0.8609 (<0.0001)	0.0%	875.0%	650.00%	15.8%	92.5%
Isolation	63	63.5% (40)	93.0%	100%	0.8944 (<0.0001)	0.0%	900.0%	650.0%	18.4%	100%

Notice that antibody ELISA outperforms cellular tests by over 700% complementary sensitivity but is consistent with post-mortem pathology and isolation detection. CITT, Comparative Intradermal Tuberculin Test; N, Number of sheep tested; Prevalence, Apparent prevalence according to the test; SE, Sensitivity according to the final post-mortem infection status; Sp, Specificity according to the final post-mortem infection status; kappa, Cohen’s agreement coefficient; p, kappa probability due to chance; Complementary Sensitivity, Ratio between the number of positives only in the row test and the total number of positives in the column reference estimate; Tuberculosis, Tuberculosis infection status after post-mortem pathological and microbiological examination; IGRA, Interferon-γ release assay; ELISA, Combined anti-*M. bovis* specific antibody test; + predictive, Predictive value of a positive result; ELISA 1, 2, 1&2, Anti-*M. bovis* specific antibody test in each round and in combination; Pathology, Tuberculosis-like gross lesions in the post-mortem examination; Isolation, *M. caprae* isolation in the post-mortem examination.

Interestingly, all sheep had very good body condition and 24 out of 44 sheep with confirmed infection had TB lung lesions (54.5%). *C. pseudotuberculosis* was isolated from one animal that was negative in the ELISA, and whose gross lesions in lung were histologically compatible with pseudotuberculosis, but that yielded *M. caprae* from the tracheobronchial lymph node. Most of the lesions were observed in lymph nodes (61.9%), while only 36.5 and 7.9% from lung and viscera, respectively. Lesions were very similar to those seen in typical bovine TB: multiple coalescing necrotic foci of a pale-yellow color with intense mineralization and fibrosis both in lymph nodes and in lungs. No significant differences were found according to breed (FET *p* = 0.3023) or sex in the first ELISA, but adults had a significantly higher seroprevalence (16.9% vs. 5.9%; *p* = 0.0051). In the anamnestic ELISA, breed and age differences were highly significant (Table 2), but not sex. Taken jointly, only the difference between adults (52.0%) and lambs (11.9%) was statistically significant (*p* < 0.0001).

All goats were negative to all the diagnostic tests performed (ST, IGRA, ELISA, and pathological and microbiological methods).

The flock was the last one in the village to use the traditional management system of daily walking from the farm to the surrounding fields with crop residues and spontaneous grass between harvests. Feed was purchased from registered producers and straw from the local producers. Sheep and goats spent the days together *en route* and grazing but were housed in separate barns for the nights. Average replacement rate during the last 4 years was 38.1% for sheep and 18.7% for goats, with similar culling rates. Sheep breeders were introduced in the flock from the same or a neighboring province in 2018 (10 from same province, nine from neighboring province), 2019 (38 from neighboring province), and 2020 (15 from same province, one from the same neighboring province). A total of six goats were introduced from same province in 2021. A TB outbreak occurred in a goat farm in the same municipality in 2014 caused by *M. caprae* spoligotype SB0416 and caused its end. Drinking points were negative for MTC.

## Discussion

Routine slaughterhouse inspection allowed detection of unapparent MTC infection in a sheep-goat mixed flock. This underlines the importance of this type of surveillance (23), which largely depends on the good performance of public health veterinarians and stresses the importance of collaboration between them and animal health services (24–26). Our results further reinforce the need to allocate the resources that secure an efficient performance of this surveillance method (27, 28). It must be noticed that the index case was a routinely culled ewe because of production, not clinical reasons, from a mixed flock with a goat population that had undergone annual TB skin testing in the past 8 years before (last test on 31 August 2020), always with negative results. The commercial antibody ELISA used in this case allowed detection of 82.5% of culture-positive sheep out of the 63 that were necropsied (index case was not tested through ELISA). The figure was slightly lower if positives to either culture or pathology were used as the reference (81.4%). ST only detected 9.3% of positives to either of these two reference methods and IGRA only 12.5%. This may look surprising since humoral immunity tests have been considered to have low sensitivity in cattle (29, 30). In spite of scientific evidence pointing out that it could very specifically complement the sensitivity of cellular immunity-based tests (detection of up to an additional 33% infected animals not identified by cellular tests) (28, 30), its use has not been incorporated into cattle control programs yet (29, 31). The only other large study of TB in sheep showed similar high prevalence rates between isolation (50.4%), histopathology (83.2%), and ELISA (59.0%), but much lower for IGRA (4.9%) and ST (24.9%) with highly concordant rates of sensitivity (93.5 to 100%) for the former three. Although the sensitivity and specificity calculations were based on a small number of cases (23 and 31, respectively), the low specificity rates (37.5–50.0%) found suggest that many truly infected cases might have failed to be detected rather than being false positives and, therefore, likely indicating that the real prevalence was substantially higher (6). Qualitative and quantitative analyses indicate that infection increases with age. That might explain why the antibody ELISA missing in a few cases could have been due to low antibody levels in recent infections. In general, this pattern of immune diagnostic response must be seriously taken into account because it means that the official cellular diagnostic techniques for TB diagnosis in cattle and goats might be dramatically failing to identify a potential hidden MTC reservoir in an important and well managed domestic species but not submitted to periodic controls for the detection of infection. If we add the recent TB findings in free-ranging swine (32–36), it becomes evident that it is urgent to include non-cattle domestic species in eradication programs taking advantage of new improved humoral TB diagnostic protocols. While these insights do cover all angles in such a costly and advanced eradication program focused on a single species, it cannot be overlooked that this outbreak was caused by a totally new spoligotype that might have specific pathogenic and epidemiological particularities such as low virulence and high sheep adaptation. Actually, the failure of the epidemiological investigation to point out an origin suggests that the bacteria might have been evolving in this sheep flock acquiring new clinicopathological and epidemiological features that could eventually require a higher taxonomical status. However, lesions did not show any pathological trait that could be attributed to a differential particularity of this specific strain. Further characterization of the strain (e.g., whole genome sequencing) might shed light in this sense.

In general terms, cellular tests have proven to be much more sensitive than humoral ones for the diagnosis of bovine TB in cattle submitted to periodical TB eradication programs. However, these results confirm our own unpublished experience in cattle that antibody tests could complement cellular ones for the detection of residual/anergic individuals, because the former detect fewer cases but still have a high complementary sensitivity for identifying residual infections that the cellular tests are missing. These cases might explain why in Spain, up to 25.3% of bovine TB-infected herds are attributed to residual internal or undetected external infection according to the last report of the Spanish Ministry of Agriculture on bovine TB (37) that also confirms an earlier observation reporting a 22.3% (28). The skin test has been in service for over one century and has allowed resonant success, but current evidence that a few infected individuals still go undetected in spite of the recurrent and huge administrative and economic investments should show us the way to take a new approach. Knowing that humoral tests provide an additional sensitivity that is close to the observed rates of residual and imported new TB outbreaks, it seems clear that a breakthrough is necessary. Contrary to other studies, in this case cellular tests, including IGRA, have much lower sensitivity (about one third) than the ELISA that, actually, had a complementary sensitivity of 7.0% relative to combined pathology and isolation. In a recently published Irish study (38) reporting 30.7% of cattle negative to the official comparative test although positive to a highly specific commercial antibody test with a WOAH homologated specificity of 99.7% (39), authors suggest that the serological results might relate to intensive skin testing. However, it could also be related to a limited sensitivity of the ST when residual/anergic infection (not detected by ST) is more significant than expected.

There is another striking finding of this study that goes against generally accepted knowledge on TB. It is that infection was highly prevalent in sheep, but not in goats. Goats have long been recognized as a host of MTC infections and have even led to name the bacterial species *M. caprae* (40–42). The fact that in this mixed herd all goats had been negative to repeated testing and still were negative to all tests when sheep had such a high prevalence is indeed an extraordinary finding that challenges previous understanding of TB epidemiology in domestic ruminants and must be considered in the future planning of bovine TB control programs. Sheep cannot be any longer ignored in the epidemiology of TB, which should not be defined as bovine anymore (11).

Regarding post-mortem inspection, 54.5% of tuberculous sheep had TB lesions in lungs. That is, about half of the infected animals had lesions only in lymph nodes that required an exhaustive post-mortem examination. The rough similarity of TB lesions with caseous lymphadenitis, together with the assumption that ovine TB is rare, is likely to have heavily contributed to overlooking sheep TB.

This silent pattern of TB infection in sheep reported here, building on previous studies indicating an unsuspected high prevalence in mixed species farms (6), raises fears that a huge reservoir of infection in a domestic species might have been worldwide overlooked by a technical issue. Addressing it by extending the use of humoral tests is urgent to help neutralize the Trojan horse-like impact of hidden MTC infections on bovine TB control programs.

Failure to use the cheaper and more complementarily sensitive antibody tests for residual infection detection could make impossible to effectively eradicate bovine TB in many countries once arrived at its last stages. Given the effectiveness of slaughterhouse passive surveillance, complementing visual inspection with systematic (all animals, a percent, or only those from suspect or unknown TB status farms or counties depending on the epidemiological status and resources availability) application of the relatively unexpensive serological tests could become a powerful tool for tracing undetected TB-infected farms. We think that in a moment in which the European TB eradication programs have reached substantial regional eradication, the recognition of ELISA as an official test both for cattle and for other TB-susceptible species and generalizing its use in active TB infection detection should be very effective in reducing exposure risks to humans and cattle. In summary, after acknowledgment of the complex epidemiology determined by cattle sharing environments with other TB-susceptible species like wild and domestic ruminants, rabbits (43) wild boars, and even badgers (41, 42, 44–50), adapting diagnostic methods to specific species epidemiology and pathogenesis could more efficiently lead to faster TB control.

## Data availability statement

The datasets presented in this study can be found in online repositories. The names of the repository/repositories and accession number(s) can be found at: https://www.visavet.es/mycodb/index-en.php, SB2737.

## Ethics statement

Ethical approval was not required for the studies involving animals in accordance with the local legislation and institutional requirements because this study was carried out as a part of an EU-Spain approved Animal Health control program carried out by the local Animal Health and Welfare authority. Written informed consent was obtained from the owners for the participation of their animals in this study.

## Author contributions

RJ: Conceptualization, Formal analysis, Investigation, Methodology, Writing – original draft, Writing – review & editing. LF-V: Investigation, Methodology, Writing – review & editing. MF: Investigation, Methodology, Writing – review & editing. IF: Data curation, Investigation, Methodology, Writing – review & editing. GC: Investigation, Methodology, Writing – review & editing. MG: Investigation, Writing – review & editing. JG: Conceptualization, Data curation, Funding acquisition, Investigation, Methodology, Project administration, Writing – review & editing. IS: Conceptualization, Data curation, Formal analysis, Funding acquisition, Investigation, Methodology, Project administration, Writing – review & editing.
